# Interactions between Genetics and Sugar-Sweetened Beverage Consumption on Health Outcomes: A Review of Gene–Diet Interaction Studies

**DOI:** 10.3389/fendo.2017.00368

**Published:** 2018-01-08

**Authors:** Danielle E. Haslam, Nicola M. McKeown, Mark A. Herman, Alice H. Lichtenstein, Hassan S. Dashti

**Affiliations:** ^1^Nutritional Epidemiology Program, Jean Mayer U.S. Department of Agriculture Human Nutrition Research Center on Aging, Tufts University, Boston, MA, United States; ^2^Division Of Endocrinology, Metabolism, and Nutrition, Department of Medicine, Duke University School of Medicine, Durham, NC, United States; ^3^Cardiovascular Nutrition Laboratory, Jean Mayer U.S. Department of Agriculture Human Nutrition Research Center on Aging, Tufts University, Boston, MA, United States; ^4^Center for Genomic Medicine, Massachusetts General Hospital, Boston, MA, United States; ^5^Program in Medical and Population Genetics, Broad Institute, Cambridge, MA, United States

**Keywords:** carbohydrate metabolism, observational studies, genetics, diet, type 2 diabetes, sugar-sweetened beverages

## Abstract

The consumption of sugar-sweetened beverages (SSB), which includes soft drinks, fruit drinks, and other energy drinks, is associated with excess energy intake and increased risk for chronic metabolic disease among children and adults. Thus, reducing SSB consumption is an important strategy to prevent the onset of chronic diseases, and achieve and maintain a healthy body weight. The mechanisms by which excessive SSB consumption may contribute to complex chronic diseases may partially depend on an individual’s genetic predisposition. Gene–SSB interaction investigations, either limited to single genetic loci or including multiple genetic variants, aim to use genomic information to define mechanistic pathways linking added sugar consumption from SSBs to those complex diseases. The purpose of this review is to summarize the available gene-SSB interaction studies investigating the relationships between genetics, SSB consumption, and various health outcomes. Current evidence suggests there are genetic predispositions for an association between SSB intake and adiposity; evidence for a genetic predisposition between SSB and type 2 diabetes or cardiovascular disease is limited.

## Introduction

Sugar-sweetened beverages (SSBs), such as sodas, fruit-flavored drinks, and sports drinks, are a significant source of dietary added sugars and a major contributor to excess energy intake (1). Global averages of SSB consumption range up to one 8-ounce serving/day (2), and contribute between 3 and 10% of daily energy consumption (3–6). Observational data suggest that higher SSB consumption is linked to a host of chronic diseases, including cardiovascular disease (CVD), type 2 diabetes (T2D), obesity, non-alcoholic fatty liver disease (NAFLD), and gout (7–11). Consequently, dietary guidance consistently recommends limiting added sugar consumption, particularly from SSBs (1, 12, 13). Although secular trends in dietary behavior suggest a decline in SSB consumption in recent years, national surveys suggest that >50% of US and European youth and adults continue to consume at least one serving of SSB daily (3, 5, 6, 14–16). Thus, SSB consumption continues to be a major public health concern globally.

Observational and experimental evidence linking SSB consumption to heritable metabolic risk factors and disease risk, i.e., those with underlying genetic predispositions, have paved the way for gene–SSB interaction studies (17–19). These gene–diet interaction studies may provide insight into the molecular mechanisms by which SSB consumption influences disease risk. From a public health perspective, this knowledge could be used to develop personalized dietary recommendations for the primary prevention and treatments of chronic diseases, and may provide motivation for patients to adhere to lifestyle guidance (20). The purpose of this review is to summarize current gene–SSB interaction studies on chronic disease risk factors and disease outcomes.

## SSB Consumption, Genetics, and Health Outcomes

We conducted a comprehensive PubMed search for literature on SSB consumption and genetic interactions for publications through July 2017. Our search strategy combined terms for SSB consumption [“sugar-sweetened beverage(s),” “soda(s),” “sugar,” “beverage(s),” “drink(s)”], diet (“diet,” “dietary,” “food”), genetics [keywords: “gene(s),” “genome,” “genetic”], and/or interactions [“interaction(s),” “modify(ies),” “modification(s)”]. We also cross-referenced recovered studies and relevant reviews to identify additional studies. We identified nine published studies linking SSB to a variety of health outcomes with consideration for underlying genetic predisposition (Table 1). Studies included cross-sectional and longitudinal analyses of population-based cohort studies, and meta-analytic data from population-based cohort studies. The genetic variants investigated were either limited to single loci or expanded multiple loci (Table 2).

**Table 1:** Population-based studies of the interaction between genetics and sugar-sweetened beverage (SSB) consumption on various health outcomes.

Author (reference)	Year	*n*	Region	Age, year mean (SD)	Female, %	BMI, kg/m^2^mean (SD)	Total energy intake, kcal/d mean (SD)	SSB, serving/day mean (SD)^a^	Outcomes	Key Observations
Qi (26)	2012	33,097	United States	53.2 ± 7.17	86.6	25.6 ± 4.64	1,742 ± 518	0.28 ± 0.60	4-year change in BMI, incident obesity	Significant positive interaction between SSB consumption and BMI genetic risk score on 4-year change in BMI and incident obesity (*p* < 0.001)
										The increases in BMI per increment of 10 risk alleles were 1.00 for a consumption of less than one serving per month, 1.12 for one to four servings per month, 1.38 for two to six servings per week, and 1.78 for one or more servings per day (*p* < 0.001 for interaction)
										The relative risks of incident obesity per increment of 10 risk alleles were 1.19 (95% confidence interval [CI], 0.90–1.59), 1.67 (95% CI, 1.28–2.16), 1.58 (95% CI, 1.01–2.47), and 5.06 (95% CI, 1.66–15.5) (*p* = 0.02 for interaction)

Batt (67)	2014	1,634 (*n* = 925 cases)	New Zealand (Polynesian and Caucasian)	50.2 (17–94)	34.5	32.0 (18.1–77.0)^b^	NA	NA	Gout, and serum urate levels	Significant positive interaction between SSB consumption and *SLC2A9* genotype on gout risk (*p* = 0.010), whereas among carriers of the gout-protective allele of *SLC2A9*, each extra daily SSB serving associated with a 15% increase in risk (*p* = 0.078), compared with a 12% increase in non-carriers (*p* = 0.002). The interaction term was significant in pooled (p_Interaction_ = 0.01), but not meta-analyzed (p_Interaction_ = 0.99) data. In the US cohort, with each extra daily serving, a greater increase in serum uric acid was observed among protective allele carriers (0.005 (*p* = 8.7 × 10^−5^) compared with 0.002 (*p* = 0.016) mmol/L)
		7,075 (*n* = 148 cases)	United States (Caucasian)	53.8 (44–65)	52.6	26.4 (14.4–54.6)^b^				

Nobili (57)	2014	200	Italy	11 (10,13)^c^	56.0	25.1 (22,27.4)^c^	NA	NA	Steatosis severity (%)	Significant positive interaction between consumption of SSB and *PNPLA3 I148M* genotype on severity of steatosis (*p* = 0.033)

Sonestedt (50)	2015	26,455	Sweden	57.9 ± NA	62.5	25.7 ± NA	2,280 ± NA	0.23 ± NA	Incident CVD, and TG, HDL-C, and LDL-C	No significant interactions observed between SSB consumption and outcomes (*p* > 0.05)

Brunkwall (27)	2016	26,726	Sweden	56.3 ± 7.87	62.1	25.7 ± 3.8	2,173 ± 606	0.31 ± 0.57	BMI	Significant positive interaction between SSB consumption and BMI genetic risk score on BMI (*p* < 0.05)

Olsen (28)	2016	4,765	Denmark	47.6 ± NA	50.3	NA	2,143 ± NA	0.05 ± NA	Change in body weight, waist circumference, waist-to-hip ratio regressed on BMI	Significant negative interaction between soft drink consumption and waist circumference genetic risk score on change in body weight (*p* < 0.01). Significant positive interaction between soft drink consumption and both BMI and adiposity genetic risk scores on waist circumference change (*p* = 0.001).

Zheng (51)	2016	3,311 (*n* = 1,560 cases)	Costa Rica	57.7 ± 11.7	24.5	26.2 ± 4.11	2,598 ± 862	1.79 ± 1.44	Myocardial infarction (based on WHO criteria)	Significant positive interaction between SSB consumption and per-risk allele of rs4977574 increased risk of myocardial infarction (*p* < 0.05)

										A genetic risk score derived from three SNPs in the same locus also showed a significant interaction with SSB consumption on MI risk

Hosseini-Esfahani (41)	2017	828 (*n* = 414 cases)	Iran	42.3 ± 12.5	44.0	24.5 ± 4.0	2,338 ± 1,025	NA	Metabolic syndrome (based on modified National Cholesterol Education Program/Adult Treatment panel III (ATP III) definition)	Significant positive interaction between SSB consumption and specific haplotypes at *APOA1/APOC3* loci (GA+AA rs670/CT+TT rs5069/CC rs5128 genotypes) on risk of MetS (*p* = 0.03)
										Note: when accounting for multiple comparisons, interaction no longer significant

McKeown and Dashti (37)	2017	37,748	United States, Netherlands, Finland, Denmark, Sweden Australia	55.7 ± 7.1	56.4	26.9 ± 4.44	1,994 ± 644	0.31 ± 0.67	Fasting glucose and fasting insulin	Suggestive interaction was observed between genetic variant in *KLB* (rs1542423) and SSB consumption on FI (*p* = 0.006). No other significant interactions found between selected genetic variants in CHREBP–FGF21 pathway and FG/FI.

*^a^SSB consumption was ascertained by a semi-quantitative food-frequency questionnaire alone (Qi et al., Hosseini-Esfahani et al., Zheng et al., Nobili et al., and Batt et al.,) or a combination of semi-quantitative food-frequency questionnaire or 7-day food diary (McKeown and Dashti et al., Olsen et al., and Brunkwall et al.). SSB consumption includes fruit juices in the following studies: Qi et al., McKeown and Dashti et al., Zheng et al., and Batt et al*.

*^b^Range*.

*^c^Median (interquartile range)*.

*BMI, body mass index; CVD, cardiovascular disease; FG, fasting glucose; FI, fasting insulin; HDL-C, high-density lipoprotein cholesterol; LDL-C, low-density lipoprotein cholesterol; MetS, metabolic syndrome; MI, myocardial infarction; SSB, sugar-sweetened beverages; TG, triglyceride; WHO, World Health Organization*.

**Table 2:** Health outcomes that have been associated with increased SSB consumption and have been studied in regard to SSB by gene interaction.

Health outcomes	Mapped gene	rsID	Minor allele frequency^a^	Reference
Incident obesity	32 BMI-Associated Genetic Variants	rs543874, rs1514175, rs1555543, rs2815752, rs2890652, rs887912, rs713586, rs2867125, rs13078807, rs9816226, rs13107325, rs10938397, rs4836133, rs2112347, rs987237, rs206936, rs10968576, rs3817334, rs4929949, rs10767664, rs7138803, rs4771122, rs11847697, rs10150332, rs2241423, rs7359397, rs1558902, rs12444979, rs571312, rs29941, rs3810291, rs2287019	0.03–0.49	Qi et al. 2012 (26)

Adiposity	30–33 BMI-Associated Genetic Variants	rs10938397, rs2815752, rs9816226, rs987237, rs7138803, rs713586, rs12444979, rs2241423, rs2287019, rs13107325, rs2112347, rs10968576, rs3810291, rs887912, rs13078807, rs11847697, rs2867125, rs571312, rs7359397, rs3817334, rs29941, rs543874, rs1514175, rs206936, rs1555543, rs1558902, rs4929949, rs10767664, rs10150332, rs4771122	0.04–0.48	Qi et al. 2012 (26); Brunkwall et al. 2016 (27); Olsen et al. 2016 (28)

	6 Waist-circumference-Associated Loci	rs10146997, rs1121980, rs7138803, rs12970134, rs545854, rs987237	0.08–0.48	Olsen et al. 2016 (28)

	14 Waist:Hip Ratio-Associated Loci	rs1011731, rs10195252, rs1055144, rs1294421, rs1443512, rs2605100, rs4823006, rs6784615, rs6795735, rs6861681, rs6905288, rs718314, rs9491696, rs984222	0.02–0.48	Olsen et al. 2016 (28)

	33 Adiposity-Associated Loci	rs10508503, rs10838738, rs10938397, rs10968576, rs11847697, rs12444979, rs13107325, rs1424233, rs1514175, rs1555543, rs17782313, rs1805081, rs206936, rs2112347, rs2241423, rs2287019, rs2568958, rs2890652, rs29941, rs3810291, rs4712652, rs4771122, rs4929949, rs543874, rs6013029, rs6232, rs6602024, rs713586, rs7647305, rs9939609, rs10146997, rs1121980, rs7138803	0.04–0.48	Olsen et al. 2016 (28)

Fasting glucose	*CHREBP*/*FGF21*-Related Loci	rs10819937, rs10819931, rs174546, rs838133, rs4607517, rs1260326, rs2119026, rs1542423,rs799166, rs799168, rs799160, rs11974409, rs11920090, rs11924032, rs5438, rs3820034, rs5840, rs2954029	0.05–0.45	McKeown and Dashti et al. 2017 (37)
Fasting insulin				

Metabolic Syndrome	*APOA1*	rs670, rs5069	0.19–0.29	Hosseini-Esfahani et al. 2017 (41)
	*APOC3*	rs5128		

TG	TG-associated genetic variants	rs1042034, rs4846914, rs1260326, rs2972146, rs645040, rs442177, rs9686661, rs6882076, rs2247056, rs17145738, rs11776767, rs1495741, rs12678919, rs2954029, rs2068888, rs174546, rs964184, rs11613352, rs4765127, rs2412710, rs2929282, rs1532085, rs11649653, rs3764261, rs10401969, rs439401, and rs5756931	0.02–0.47	Sonestedt et al. 2015 (50)

HDL-C	HDL-C-associated genetic variants	rs4660293, rs1689800, rs4846914, rs1042034, rs12328675, rs2972146, rs13107325, rs6450176, rs2814944, rs605066, rs17145738, rs9987289, rs12678919, rs2293889, rs2954029, rs581080, rs1883025, rs2923084, rs3136441, rs174546, rs964184, rs7941030, rs7134375, rs11613352, rs7134594, rs4759375, rs4765127, rs1532085, rs2652834, rs3764261, rs16942887, rs2925979, rs11869286, rs4129767, rs7241918, rs12967135, rs7255436, rs737337, rs4420638, rs1800961, and rs181362	0.04–0.47	Sonestedt et al. 2015 (50)

LDL-C	LDL-C associated genetic variants	rs12027135, rs2479409, rs629301, rs514230, rs1367117, rs4299376, rs12916, rs6882076, rs3757354, rs1800562, rs3177928, rs9488822, rs1564348, rs12670798, rs9987289, rs2081687, rs2954029, rs9411489, rs2255141, rs174546, rs964184, rs11220462, rs11065987, rs1169288, rs8017377, rs3764261, rs2000999, rs6511720, rs10401969, rs4420638, rs2902940, and rs6029526	0.05–0.48	Sonestedt et al. 2015 (50)

Cardiovascular disease	Lipid-associated genetic variants	(all TG, HDL-C, and LDL-C associated genetic variants)	0.02–0.48	Sonestedt et al. 2015 (50)

Myocardial Infarction	Chromosome 9p21 Loci	rs4977574, rs2383206, and rs1333049	0.41–0.50	Zheng et al. 2016 (51)

Hepatic Steatosis	*PNPLA3*	rs738409 (l148M PNPLA3)	0.48	Nobili et al. 2014 (57)

Gout	*SLC2A9*	rs11942223 (NZ population)	0.09	Batt et al. 2014 (67)

Uric Acid Concentration		rs6449173 (US population; surrogate marker)	0.22	

*^a^Minor allele frequency is based on study-specific population and ancestry*.

*BMI, body mass index; HDL-C, high-density lipoprotein cholesterol; LDL-C, low-density lipoprotein cholesterol; NZ, New Zealand; SSB, sugar-sweetened beverages; TG, triglyceride*.

### Obesity

Several observational studies have observed a positive relationship between SSB consumption and adiposity (21–23), including a recent meta-analysis which found a 0.22 kg annual weight gain per additional serving of daily SSB consumption (9). Longitudinal analyses of 1,003 participants from the Framingham Heart Study (24) observed 29% greater increase in visceral, a risk factor for T2D and CVD (25), but not subcutaneous adipose tissue, among daily consumers SSB compared with non-consumers over a 6-year period.

Evidence from three separate investigations, encompassing a total of 50,000+ participants from cohorts in the U.S. and Northern Europe and using similar analytic approaches, found that greater consumption of SSB exacerbated the association between obesity-related genes and BMI (26–28). These studies used weighted genetic risk scores aggregating over 30 obesity-related genetic variants identified from genome-wide association studies to quantify genetic predisposition to obesity (Table 2). Qi and colleagues observed consistent findings across three US cohorts whereby genetic predisposition to obesity was exacerbated with greater SSB consumption (26). In a pooled analysis of two cohorts, the increase in BMI per each 10 BMI-related risk-allele increment was 1.78 among the highest SSB consumers (≥1 serving/day), compared to only 1.00 among the lowest SSB consumers (<1 serving/month) (*P*_interaction_ = < 0.001) (26). Consistent with BMI, the relative risk of incident obesity per increment of 10 risk alleles was 5.06 (95% CI 1.66–15.5) among the highest SSB consumers (≥ 1 serving/day), compared to only 1.19 (95% CI 0.90–1.59) among the lowest SSB consumers (<1 serving/month) (*P*_interaction_ = 0.02). The study replicated the trend in a third independent cohort (26). Brunkwall and colleagues observed similar interactions in a meta-analysis of cross-sectional data from two Swedish cohorts (27). The increase in BMI, per each 10 BMI-related risk-allele increment, was 1.28 kg/m^2^ (SE = 0.17) among the highest SSB consumers compared to 0.82 kg/m^2^ (SE = 0.11) among seldom SSB consumers (*P*_interaction_ = 0.03) (27). Olsen and colleagues generated four genetic risk scores including variants associated with BMI, waist circumference, waist-to-hip ratio, and a combined score, and assessed SSB consumption in relation to annual changes in body weight, waist circumference, and waist circumference adjusted for BMI for three cohort studies (28). Consistent with previous reports, yet with a smaller effect size, higher genetic risk score exacerbated the association between SSB consumption and change in waist circumference, whereas with each risk allele increase in genetic risk score (for BMI-related variants or the complete score) the annual change in waist circumference per each additional serving of SSB per day was 0.05 cm [genetic risk score for BMI-related variants adjusted for BMI: 0.05 (0.02, 0.09) cm per year; *p* = 0.001; complete genetic risk score (variants associated with BMI, waist circumference, and waist-to-hip ratio): 0.05 (0.02, 0.07) cm per year; *p* = 0.001] higher. Interestingly, additional analyses in this study provided the first evidence that a genetic predisposition to a high waist circumference may attenuate the association between SSB consumption and body weight gain. For each waist circumference-related risk allele, change in annual body weight was lower with each additional serving of SSB per day [−0.06 (−0.10, −0.02) kg per year; *p* = 0.001].

### T2D and Metabolic Syndrome (MetS)

Evidence from ecological, cross-sectional, and prospective studies suggest that higher SSB consumption and greater consumption of added sugars are dietary exposures linked to greater T2D risk (29). Indeed, several large investigations of prospective cohort studies have corroborated these associations (8, 30–32), and were summarized in a recent meta-analysis which indicated a 13% greater risk of T2D with each additional serving of SSB consumption (32). Higher SSB consumption has also been linked to increased risk of insulin resistance (HOMA-IR) (33, 34), higher rate of impaired fasting glucose (35), and greater risk of MetS (36).

We have recently investigated how these relationships may be inconsistently associated with variants involved in fructose metabolism and the ChREBP–FGF21 pathway by selecting genetic variants that are critical determinants of hepatic glucose metabolism, regulation of ChREBP and plasma TG concentrations, or the metabolic hormone *FGF21* and its obligate receptor beta-klotho (*KLB*) (37). In a meta-analysis of 11 cohorts from the Cohorts for Heart and Aging Research in Genomic Epidemiology (CHARGE) Consortium including up to 34,748 adults of European descent, we first replicated earlier observations that higher SSB consumption was positively associated with fasting insulin and glucose concentrations. In addition, we identified a suggestive interaction between a genetic variant (rs1542423) in the *KLB* locus on fasting insulin. However the interaction finding did not replicate upon further investigation in replication cohorts. In a small case-control study, Hosseini-Esfahani and colleagues also investigated the potential interaction between SSB consumption and three genetic variants (rs670, rs5069, and rs5128) in the *APOA1/APOC3* locus, previously associated with dyslipidemia (38–40), on prevalence of MetS in approximately 800 residents of Iran (41). Haplotype analysis identified an interaction between SSB consumption and the combination of two genotypes at the locus of interest (GA+AA rs670/CT+TT rs5069/CC rs5128) on MetS risk (*P*_interaction_ = 0.03). Carriers of the genotype and those who were the highest consumers of SSB had nine times higher odds of MetS compared to those with the same genotype but were the lowest SSB consumers. Among those with other combinations of genotypes, the observed increase in MetS risk with higher SSB consumption was blunted. Given the large number of statistical tests, the selected 2-sided *p*-value threshold of <0.05 applied in this study is not sufficiently conservative.

### Cardiovascular Disease

Mounting evidence suggests a link between SSB consumption and CVD (19). Each additional serving of SSB consumption was estimated to associate with a 22% increased risk for myocardial infarction (7) and a 13% increased risk for stroke (42). Consistent with those results, higher SSB consumption associates with intermediate CVD risk factors including obesity (9, 21–23), hypertension (43–45), and dyslipidemia (35, 46–49). These associations prompted initial efforts to test whether genetic variation might interact with SSB consumption to influence CVD risk. One prospective cohort study in 26,455 Swedish adults investigated whether genetic risk for dyslipidemia [weighted genetic risk score for 80 known genetic variants associated with triglyceride (TG), high-density lipoprotein cholesterol (HDL-C), or low-density lipoprotein cholesterol (LDL-C) concentrations] interacted with SSB consumption to influence incident ischemic CVD and plasma lipid concentrations (TG, HDL-C, and LDL-C). The study did not observe significant interactions (50). By contrast, another study took a candidate gene approach for a locus on chromosome 9p21 famously known for its robust association with CVD. In 3,311 Hispanic adults, Zheng and colleagues observed a 48% increased risk of myocardial infarction per each risk allele of rs4977574 (in the 9p21 region) in participants with high SSB consumption (>2 serving/day) than in those with low SSB consumption (<1 serving/day) (*P*_interaction_ = 0.005) (51). No significant interactions were observed for the two other variants tested from the same region (rs2383206 and rs1333049).

### Non-Alcoholic Fatty Liver Disease

Evidence also suggests a positive relationship between SSB consumption and NAFLD (52). In a cross-sectional study of 2,634 U.S. adults, daily SSB consumers were found to be 1.5 times as likely to have NAFLD compared to SSB non-consumers (10). The strongest genetic determinant of NAFLD is the *PNPLA3* locus (53, 54), which has been consistently associated with increased liver fat synthesis (steatosis) in genome-wide association studies (54–56). In a sample of 200 Italian youths (10–13 years) at high risk for NAFLD, Nobili and colleagues examined whether *PNPLA3* interacted with SSB consumption to influence the severity of hepatic steatosis (57). They observed that the *PNPLA3* genetic variant was more strongly associated with severity of hepatic steatosis among those reporting drinking SSB at least once weekly compared to less frequent consumption (*p* = 0.03).

### Gout

Gout is a disease in which elevated circulating uric acid may crystallize, and these crystals deposit in joints, tendons and surrounding tissues, causing inflammation and joint pain. The disorder has been associated with increased CVD risk (58). SSB consumption associates with risk for gout in both observational and experimental studies (59–63). Batt and colleagues investigated whether SSB consumption may differentially influence gout risk and serum uric acid levels with variants in the *SLC2A9* locus among 1,634 Polynesian and Caucasian individuals living in New Zealand (N.Z.) [a region with higher prevalence of gout (64)] and 7,075 Caucasians living in the U.S. *SLC2A9* encodes the GLUT9 facilitative transporter which is a high affinity transporter for uric acid (65). The *SLC2A9* variant of interest is known to explain ~4% of the variance in serum urate levels (66, 67). Upon pooling individual level data from the N.Z. and U.S. cohorts, with each additional daily SSB serving there was a 12% increased gout risk among non-carriers of the protective C allele, whereas no increase in risk among carriers of the gout-protective C allele (*P*_interaction_ = 0.01). No statistically significant interactions were observed for plasma uric acid levels. In the U.S. cohort there was a trend (*P*_interaction_ = 0.062) toward higher serum uric acid levels with each additional daily SSB serving among carriers of the gout-protective C allele [change in serum urate per increase in category of SSB intake (95% CI): 0.005 (0.003–0.007) mmol/L].

## Discussion

In this review, we have summarized the limited body of evidence describing how genes implicated in various diseases may interact with SSB consumption to modify cardiometabolic health and chronic disease risk. To date, the strongest evidence for interaction between genes and SSB consumption is for obesity in three independent studies that consistently support a link between a genetic risk for obesity and SSB consumption on changes in adiposity (26–28). The majority of current evidence suggests that the adverse effects of SSB consumption on health may be strongest among subgroups with a genetic predisposition for the adverse metabolic outcomes, with some exceptions. In two studies, one related to changes in body weight (28) and one related to gout (67), the genetic predisposition seems to attenuate or possibly mask the association between SSB intake and the outcome.

The public health implications of these findings should be carefully interpreted both in context of the effect size of the interaction and in the frequency of the effect variant. For impactful personalized nutrition, the effect of the interaction should be clinically meaningful and the frequency of the effect variant common among the population. Such an interaction may be helpful in serving as a motivational tool to encourage compliance with guidance on lifestyle modification for select individuals (20). In the current review, effect sizes of interactions ranged from larger interactions with likely impactful effect sizes (26, 51) to smaller effect sizes (28, 37). While interactions with smaller effect sizes may have unclear clinical and translational impact, they may provide unique biological insight and help frame subsequent research questions.

Inferring potential mechanisms by which genetics and SSB consumption may influence disease onset is more feasible in interaction studies involving genetic variants with known functions. Batt and colleagues present an example of a gene by SSB consumption interaction study with the potential to provide insight into the molecular origin of increased risk of gout for some individuals (67). It is known that *SLC2A9* encodes the GLUT9 transporter, a renal uric acid transporter (59, 65, 68), and genetic variants of the transporter associate with blood levels of uric acid and risk of gout (66, 69–71). SSB are composed of 50% fructose, and a major end product of fructose metabolism is uric acid. Genetic variants associated with a reduced ability to clear the increased uric acid produced during fructose metabolism may be one mechanism by which increased SSB consumption could lead to increased blood levels of uric acid in some individuals (59, 72). Thus, it is possible that individuals with a *SLC2A9* mutation that leads to a defective GLUT9 transporter may have decreased ability to eliminate high uric acid loads produced from high-fructose metabolism following high SSB consumers. Thus, these individuals may generally have higher blood uric acid levels, so even a small increase in mean uric acid levels may lead to an increase in gout risk. Potential for mechanistic insights such as this cannot be determined from studies of genes with limited knowledge of their biological function or a host of pleiotropic genes.

There are general differences in the methods used to select genetic variants among the studies described here. Single genetic variants previously known to be strongly associated with the outcomes of interest from genome-wide association studies were selected for T2D, CVD, NAFLD, and gout, while an aggregate score, also related to the outcome, was selected for obesity and MetS. In our research with respect to glycemic traits (37), we have selected genetic variants related to both the exposure and outcome. In the case of the adiposity genetic risk score analyses, as the functionality of several BMI-related loci remain largely unknown, it is difficult to pinpoint mechanisms by which the score aggregating several of these variants interacts with SSB consumption. Thus, individual variant interaction tests may be insightful as single variants can be more readily mapped to a biological function. Both Qi and colleagues (26) and Brunkwall and colleagues (27) conducted follow-up analyses and provided evidence for modest effects of each individual variant by SSB consumption interaction on BMI. While nominal significance was observed for a few individual SNPs [Qi and colleagues: rs543874 in *SEC16B* (*p* = 0.0003) and Brunkwall and colleagues: rs1555543 in *PTBP2* (*p* = 0.02)], these findings do not show significance when accounting for multiple testing. On the contrary, Olsen and colleagues provide evidence that *GPRC5B* rs12444979 individual interaction with SSB consumption for change in waist circumference, which warrants further follow-up. Current knowledge regarding genetic variants related to variability in SSB consumption is limited. A genome-wide association analysis of macronutrient intake has identified one locus that was associated with carbohydrate intake (73). However, efforts to disclose genetic variants related to single foods and beverages are ongoing and are made possible by large biobanks with genome-wide genetic data and dietary intake data (https://biobankengine.stanford.edu/coding/INI1309).

Direct-to-consumer genetic testing companies that offer nutrition recommendations currently do not offer personalized recommendations related to SSB consumption as they do not account for the interactions related to SSB consumption described in this Review. While they may not include these recommendations simply because of the greater risk of chronic diseases with higher SSB consumption in the general population, it may also, in part, be driven by various limitations in the current evidence. With the exception of obesity, the described studies are of limited sample sizes and have not been replicated. For example, the studies related to NAFLD and MetS had modest sample sizes of 200 and 828, respectively. In the case of obesity, effort has been placed on replicating interaction findings first reported by Qi et al. in non-U.S. populations and despite minor differences in findings, the consistency of the results across longitudinal studies with multiple sampling of diet increased confidence in the finding. Attempting replication of other recent interaction findings in larger and more diverse populations, with prospective designs, is warranted to corroborate initial findings in this field as has been recently conducted for T2D (74). For the purpose of generalizability of findings, replication attempts should include populations of different age groups to account for variability in SSB consumption with age (i.e., higher consumption among younger individuals) (26, 67), and cohorts from various countries to account for differences in SSB formulation (i.e., Europe SSB are sweetened with sucrose, which is composed of 50% fructose and 50% glucose, whereas a higher proportion of SSB in the U.S. are sweetened with high-fructose corn syrup composed of 55% fructose and 45% glucose). Other considerations for future research include differences in the exposure, SSB consumption, both in terms of dietary assessment methodology (i.e., semi-quantitative food-frequency questionnaire or 7-day food records) and in SSB consumption definition (i.e., inclusion of fruit juice). Including fruit juice in SSB definition may contribute to different results (51).

In summary, the detrimental effects of SSB consumption on disease risk is of public health concern, regardless of genetic predisposition. Imamura and colleagues estimated that SSB consumption could contribute to 1.8 million T2D events in the US over 10 years, and to 2.6 million events in the UK, even after accounting for obesity status (32). The gene by SSB consumption interaction data to-date are interesting and may suggest that some individuals who consume greater SSB may be more susceptible to greater risk of adiposity; however, further studies are needed on the effect of sugars in those with genetic predisposition for diabetes or CVD. In the meantime, there is a continued need to develop public health interventions that reduce the consumption of SSB globally.

## Author Contributions

HD contributed to the conception and design of the research; and DH and HD contributed to the acquisition, analysis, and interpretation of the data. All authors drafted the manuscript, critically revised the manuscript, agreed to be fully accountable for ensuring the integrity and accuracy of the work, and read and approved the final manuscript.

## Conflict of Interest Statement

The authors declare that the research was conducted in the absence of any commercial or financial relationships that could be construed as a potential conflict of interest. The handling editor declared a shared affiliation, though no other collaboration, with one of the authors HD.

## Abbreviations

CVD, cardiovascular disease; HDL-C, high-density lipoprotein cholesterol; LDL-C, low-density lipoprotein cholesterol; NAFLD, non-alcoholic fatty liver disease; SSB, sugar-sweetened beverages; TG, triglyceride; T2D, type 2 diabetes.
